# Functional Complement Analysis Can Predict Genetic Testing Results and Long-Term Outcome in Patients With Complement Deficiencies

**DOI:** 10.3389/fimmu.2018.00500

**Published:** 2018-03-21

**Authors:** Štefan Blazina, Maruša Debeljak, Mitja Košnik, Saša Simčič, Sanja Stopinšek, Gašper Markelj, Nataša Toplak, Peter Kopač, Breda Zakotnik, Marko Pokorn, Tadej Avčin

**Affiliations:** ^1^Department of Allergology, Rheumatology and Clinical Immunology, Children’s Hospital Ljubljana, University Medical Centre Ljubljana, Ljubljana, Slovenia; ^2^Department of Special Laboratory Diagnostics, Children’s Hospital Ljubljana, University Medical Centre Ljubljana, Ljubljana, Slovenia; ^3^University Clinic of Pulmonary and Allergic Diseases Golnik, Golnik, Slovenia; ^4^Department of Internal Medicine, Faculty of Medicine, University of Ljubljana, Ljubljana, Slovenia; ^5^Faculty of Medicine, Institute of Microbiology and Immunology, University of Ljubljana, Ljubljana, Slovenia; ^6^Department of Paediatrics, Faculty of Medicine, University of Ljubljana, Ljubljana, Slovenia; ^7^Department of Infectious Diseases, University Medical Centre Ljubljana, Ljubljana, Slovenia

**Keywords:** complement deficiency, primary immunodeficiency, laboratory analysis, genetic analysis, clinical manifestations

## Abstract

**Background:**

Prevalence of complement deficiencies (CDs) is markedly higher in Slovenian primary immunodeficiency (PID) registry in comparison to other national and international PID registries.

**Objective:**

The purposes of our study were to confirm CD and define complete and partial CD in registered patients in Slovenia, to evaluate frequency of clinical manifestations, and to assess the risk for characteristic infections separately for subjects with complete and partial CD.

**Methods:**

CD was confirmed with genetic analyses in patients with C2 deficiency, C8 deficiency, and hereditary angioedema or with repeated functional complement studies and measurement of complement components in other CD. Results of genetic studies (homozygous subjects vs. heterozygous carriers) and complement functional studies were analyzed to define complete (complement below the level of heterozygous carriers) and partial CD (complement above the level of homozygous patients). Presence of characteristic infections was assessed separately for complete and partial CD.

**Results:**

Genetic analyses confirmed markedly higher prevalence of CD in Slovenian PID registry (26% of all PID) than in other national and international PID registries (0.5–6% of all PID). Complement functional studies and complement component concentrations reliably distinguished between homozygous and heterozygous CD carriers. Subjects with partial CD had higher risk for characteristic infections than previously reported.

**Conclusion:**

Results of our study imply under-recognition of CD worldwide. Complement functional studies and complement component concentrations reliably predicted risk for characteristic infections in patients with complete or partial CD. Vaccination against encapsulated bacteria should be advocated also for subjects with partial CD and not limited to complete CD.

## Introduction

Complement is a cascade of soluble proteins and membrane expressed receptors and regulators, playing a key role in host homeostasis, inflammation, and defense against pathogens. The complement cascade can be activated by classical, alternative and lectin pathways leading to activation of anaphylatoxins C3a and C5a, opsonins C3b and C5b. Deposition of C5b onto a target initiates membrane attack complex (consisting of C5–C9 components) formation and target lysis (1). It is well recognized that homozygous mutations in complement component C1–C9 genes severely reduce complement activity and lead to recurrent infections with encapsulated bacteria and autoimmune phenomena (2, 3). However, the expressivity of complement deficiencies (CDs) could range from absence of clinical manifestations to death because of bacterial meningitis, pneumonia or sepsis in C1–C9 deficiencies and life-threatening edema in hereditary angioedema (HAE) (4).

According to published data, heterozygous mutations in complement component C1–C9 genes partially reduce complement activity that only exceptionally leads to clinical manifestations (5). However, the correlation between homozygous and heterozygous states, complement activity and clinical manifestations in a large cohort has not been studied yet. Published data are limited to small case series and 1 multicenter study evaluating clinical manifestations in 77 patients with CD (excluding HAE, MBL deficiency, and CD carriers) from 18 cities across Europe (6). However, CD was genetically confirmed in only 25% of included patients and the study did not evaluate the correlation between complement activity and clinical manifestations.

We present data from the largest single center cohort of patients with primary CD (homozygous or compound heterozygous mutation in C1–C9 genes and HAE) and CD carriers (heterozygous mutations in C1–C9 genes), including demographic data, genetic characteristics, complement activity, and clinical manifestations. The study was designed as long-term cohort study specifically addressing the correlation between homozygous and heterozygous states and complement activity, the impact of complete and partial CD on the long-term outcome in patients with CD (excluding HAE), correlation between specific mutations in *C2* (including 2 novel mutations) and complement activity, as well as prevalence and clinical manifestations of HAE.

## Materials and Methods

### Study Design

The study design was a national cohort study of all CD patients (excluding MBL deficiency and atypical hemolitic uremic syndrome without genetic defect in complement factors) included in the Slovenian national primary immunodeficiency (PID) registry and carriers of CD. The Slovenian national PID registry was established in 2007 and compiles data of all patients with PID evaluated at the secondary and tertiary care hospitals in Slovenia since January 1977. The methodology of the registry was described elsewhere (7). See supplementary material for detailed description of methods.

In Slovenia, complement activity is routinely measured in most patients after the first infection with *Neisseria meningitidis* and after recurrent infections with other encapsulated bacteria (*Streptococcus pneumoniae and Haemophilus influenzae* type b) after neonatal period or autoimmune diseases, especially connective tissue diseases and glomerulonephritis. CDs (except HAE) were diagnosed in case of persistently reduced complement activation and reduced concentration of complement component in the absence of signs of complement consumption [e.g., in systemic lupus erythematosus (SLE); except in patients with SLE with confirmed genetic defect in the complement system] or reduced complement production (e.g., liver disease). The diagnosis of HAE was established in the presence of at least one major clinical criterion (cutaneous angioedema, abdominal pain, laryngeal edema) and one laboratory criterion (reduced C1 inhibitor concentration, reduced C1 inhibitor function, or identified mutation in C1 inhibitor or FXII) as proposed in guidelines for diagnosis of HAE (8, 9).

Prevalence of CD and prevalence of all PID in Slovenia were calculated and compared to prevalence of CD in other national registries and the European Society for Immunodeficiencies (ESID) registry. Demographic characteristics and clinical manifestations leading to diagnosis were analyzed. In patients with HAE, clinical severity score was calculated based on the age of disease onset, number of organs affected and need for long-term prophylaxis and expressed with values from 0 to 10 (10). Data on mortality related to CD (death because of HAE attack or severe infection in other CD) in registered patients or their family members were analyzed.

### Study Participants

Two hundred fifty-seven patients with 51 different PID were registered in the Slovenian PID registry between January 1977 and January 2017 (152 male, 105 female; 22% adults). Seventy-five (29%) patients were diagnosed in the last 5 years. Sixty-eight patients with six different CD (HAE, C2, C6, C7, C8, and properdin deficiency) were registered in the Slovenian PID registry during the study period (33 male, 35 female, 73% adults), which represented 26% of all registered PID patients. Clinical data on 17 patients with HAE were published before (11), in the present article the data are supplemented with patients diagnosed after year 2012. At the time of the study, the majority of patients with HAE were adults (96%—23 of 24 patients); median age 49 years (6–90 years), which contrasts with other CD where adults represented 61% of the enrolled patients (27 of 44 patients). Average age at the first manifestation of complement component deficiency was 2.9 years (0.1–24 years; data available for 17 patients), whereas in HAE, it was higher, 17 (3–59) years. Average delay in the diagnosis of CD and HAE were 1.4 (0–12) years and 8.5 (6–36) years, respectively.

The genetic defect in the complement system was confirmed in 23/24 (96%) patients with HAE and in 20/44 (45%) of patients with other CD, respectively (24 patients were not genetically evaluated, because DNA or consent for genetic testing were not available). Genetic testing confirmed homozygous or compound heterozygous mutation in all subjects in whom C2 or C8 deficiency was previously diagnosed with functional tests. Of 44 patients with CD other than HAE, 20 had C8 deficiency, 18 had C2 deficiency, 2 had C6 deficiency, 2 had C7 deficiency, 1 had combined C6/C7 deficiency, and 1 had properdin deficiency. Of 24 patients with HAE, 17 patients from 9 families were diagnosed with HAE type I and 7 patients from 4 families with HAE type II. We also identified three patients in one family with HAE with normal C1 inhibitor and mutation in FXII.

Besides 68 patients with CD, we identified 20 carriers of CD (16 carriers with mutation in the *C2* gene and 4 carriers with mutation in the *C8* gene) and 4 subjects with complement levels characteristic for carrier state but without confirmed genetic mutation. Most carriers were diagnosed during evaluation of family members of patients with CD; however in two participants, carrier state was diagnosed during evaluation of suspected PID. Clinical data of 16 patients with complement component deficiency diagnosed before the year 1995 were not available, these participants were included only in the epidemiological analysis but not in the analysis of associations between complement activity and clinical manifestations.

All patients or their legal guardians gave informed consent for data collection, which was approved by the National Medical Ethics Committee. The study has been carried out in accordance with the Declaration of Helsinki.

### Correlation Between Homozygous/Heterozygous Mutations in CD (Excluding HAE) and Functional Complement Tests and Concentrations of Affected Complement Components

In the first part of the study, we analyzed if it is possible to predict genetic status of *C2* and *C8* with functional complement studies by comparing results of functional complement tests (CH50, AH50) and concentrations of C2 and C8 complement components between the following:

(a) patients with homozygous or compound heterozygous mutations causing complement component deficiency and
(b) carriers of heterozygous mutation causing complement component deficiency.

Following analyses were performed:

(a) CH50 in all subjects with C2 deficiency and in all subjects with C8 deficiency and
(b) AH50 in all subjects with C8 deficiency.

In C2 deficiency, only CH50 was analyzed as AH50 is not affected by C2. The number of subjects with deficiencies of other components of complement were too small for statistical analysis; therefore, they were presented in a descriptive manner.

### Impact of Reduced Complement Activity on Long-Term Outcome

In the second part of the study, we analyzed impact of reduced complement activity (HAE patients excluded) on long-term outcome by comparing subjects with different complement levels:

(a) complete CD: CH50 below the lowest CH50 in carriers of mutation causing C2 or C8 deficiency or AH50 below the lowest AH50 in C8 deficiency and
(b) partial CD: CH50 above the lowest CH50 in carriers of mutation causing C2 or C8 deficiency or AH50 above the lowest AH50 in C8 deficiency.

Results of functional complement tests (CH50, AH50) and concentrations of C2 and C8 complement components were compared between the following:

(a) patients with complete CD with infections;
(b) subjects with complete CD without infections;
(c) patients with partial CD with infections;
(d) subjects with partial CD without infections.

Following analyses were performed:

(a) CH50 in all subjects with CD (except 1 patient with properdin deficiency), separately for subjects with C2 and C8 deficiency;
(b) AH50 in all subjects with CD, separately for subjects with C2 and C8 deficiency;
(c) C2 in subjects with C2 deficiency
(d) C8 in subjects with C8 deficiency.

Patient samples with deficiencies of other components of complement were too small for statistical analysis (six patients with infections and one carrier without infections).

### Correlation Between Complement Activity and Specific Mutations in *C2* Gene, Including Novel Mutations (c.1754C>T and c.2024G>T)

Results of functional complement tests (CH50, AH50) and concentrations of C2 complement components were compared between the following:

(a) subjects with common homozygous deletion of 28 bp in *C2* (c.841_849 + 19del);
(b) subjects with compound homozygous mutation in *C2* (c.841_849 + 19del and c.1754C>T);
(c) subjects with compound heterozygous mutation in *C2* (c.841_849 + 19del + c.945G>C and c.1754C>T)
(d) a subject who is a carrier of mutation in *C2* (c.2024G>T).

### Statistical Methods

Average values of functional tests (CH50 and AH50) of complement activation and concentrations of complement components were calculated in all included subjects (only measurements after the first year of age were taken into account). Differences between groups were analyzed using the Student’s *t*-test for independent samples (Welch’s *t* test). Differences between groups are shown in box plots, where thick line represents the median; bottom and upper lines the 25th and the 75th percentile, respectively; and the “whiskers” the lowest and the highest values. Dotted lines represent lower and upper normal limits of the test.

## Results

**Figure 1:**
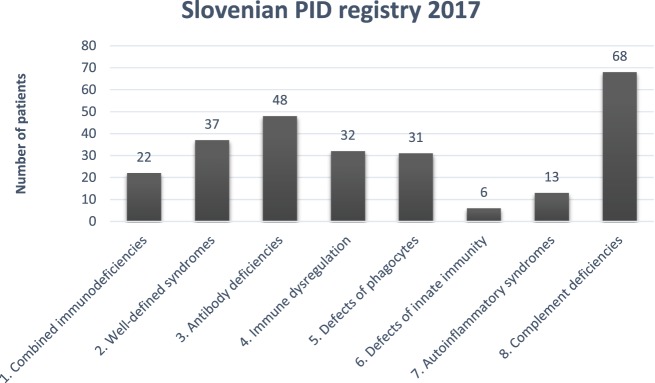
Distribution of primary immunodeficiencies (PIDs) in the Slovenian PID registry. Combined immunodeficiencies: severe combined immunodeficiency, combined immunodeficiency; well-defined syndromes: DiGeorge, ataxia-telangiectasia, hyper IgE. Wiskott-Aldrich, Nijmegen, Netherton, Schimke, osteopetrosis; antibody deficiencies: x-linked agammaglobulinemia, common variable immunodeficiency. IgA deficiency, class switch defect; immune dysregulation: autoimmune lymphoproliferative syndrome; candidiasis, ectodermal dysplasia; x-linked lymphoproliferative syndrome, familial hemophagocytic lymphohistiocytosis autoimmune lymphoproliferative syndrome, MALT1 deficiency, Chediak-Higashi, AEP2/3 deficiency; phagocytic defects: chronic granulomatous deficiency, congenital neutropenia, Papillon-Lefevre, Schwachman-Diamond, Barth; defects of innate immunity: NEMO deficiency; autoinflammatory syndromes: chronic recurrent multifocal osteomyelitis, cryopyrin-associated periodic syndrome, TNF-alpha receptor-associated periodic syndrome; complement deficiencies: C1–C9 deficiencies, hereditary angioedema, properdin deficiency.

Distribution of PID in Slovenian PID registry according to International union of immunological societies expert committee classification (12) is shown in Figure 1.

### Clinical Manifestations of Patients With CD

Only 28/44 patients with CD were evaluated for the clinical manifestations because data were not available for the 16 diagnosed before 1995. CD was diagnosed in 23 of 28 (82%) patients with CD excluding HAE after the occurrence of bacterial infections, predominantly with encapsulated bacteria (*S. pneumoniae, H. influenzae, N. meningitidis*), *Staphylococcus aureus*, and *Streptococcus pyogenes* and only in 1/28 patient after autoimmune glomerulonephritis (Table 1). CD was also diagnosed in six relatives of patients with CD, four of these had clinical manifestations consistent with CD. HAE was diagnosed in 22 of 24 patients after angioedema. Median clinical severity score in HAE was 5 (0–10), with 15 patients having peripheral edema (13 facial edema, 6 laryngeal edema, 11 abdominal pain).

**Table 1:** Clinical manifestations and genetic defects in symptomatic patients and carriers of complement deficiency excluding hereditary angioedema.

Complement def.	Mutation	Gender	Age (years)	Clinical manifestations
**Patients with C2, C6, C6/C7, C8 and properdin deficiency and clinical manifestations**				

C2 deficiency	Homozygous c.841_849 + 19del	M	16	Sepsis
C2 deficiency	homozygous c.841_849 + 19del	M	12	Recurrent pneumonia, otitis and sepsis
C2 deficiency	Homozygous c.841_849 + 19del	M	12	Recurrent pneumonia
C2 deficiency	Homozygous c.841_849 + 19del + c.945G>C	M	11	Pneumonia and recurrent sepsis
C2 deficiency	Homozygous c.841_849 + 19del + c.945G>C	M	6	Sepsis and osteomyelitis
C2 deficiency	Homozygous c.841_849 + 19del + c.945G>C	M	4	Impetigo, recurrent pneumonia and preseptal cellulitis
C2 deficiency	Compound heterozygous c.841_849 + 19del and c.1754C>T	M	5	Sepsis and nephrotic syndrome
C2 deficiency	N.A.	M	20	Purulent meningitis
C2 deficiency	Compound heterozygous c.841_849 + 19del and c.1754C>T	F	9	Recurrent otitis
C2 deficiency	Compound heterozygous c.841_849 + 19del + c.945G>C and c.1754C>T	F	5	Sepsis and recurrent pneumonia
C2 deficiency	Homozygous c.841_849 + 19del	F	7	Sepsis and preseptal cellulitis
C8 deficiency	Homozygous c1282C>T	M	57	Recurrent pneumonia and recurrent purulent meningitis
C8 deficiency	Homozygous c1282C>T	M	9	Recurrent otitis
C8 deficiency	Homozygous c1282C>T	F	28	Recurrent pneumonia and recurrent purulent meningitis
C8 deficiency	Homozygous c1282C>T	F	20	Pneumonia
C8 deficiency	Homozygous c1282C>T	F	7	Sepsis, sinusitis and recurrent otitis
C8 deficiency	Homozygous c1282C>T	F	9	Pneumonia
C8 deficiency	Homozygous c1282C>T	F	30	Purulent meningitis
C8 deficiency	Homozygous c1282C>T	F	35	Purulent meningitis
C8 deficiency	Homozygous c1282C>T	F	32	Purulent meningitis
properdin def.	N.A.	M	27	Purulent meningitis
C7 deficiency	N.A.	F	28	Recurrent sepsis
C6/C7 deficiency	N.A.	M	12	Pneumonia, recurrent otitis and recurrent herpes zoster

**Carriers of mutations causing C2 deficiency and clinical manifestations**				

Carrier of C2 def.	Heterozygous c.841_849 + 19del	M	44	Purulent meningitis
Carrier of C2 def.	Heterozygous c.841_849 + 19del	M	43	Recurrent sinusitis
Carrier of C2 def.	Heterozygous c.841_849 + 19del + c.945G>C	F	9	Recurrent impetigo
Carrier of C2 def.	Heterozygous c.2024G>T	F	4	Pneumonia
Carrier of C2 def.	Heterozygous c.841_849 + 19del	F	10	Recurrent otitis

### Clinical Characteristics of Carriers of Mutations Causing CD

Carrier state for CD (excluding HAE) was diagnosed in 22 subjects evaluated as relatives of patients with CD. Three out of 22 (14%) carriers had clinical manifestations consistent with CD: bacterial meningitis, recurrent sinusitis, and recurrent skin abscesses. Other CD carriers (19/22, 86%) have not had clinical manifestations suggestive of CD until the end of the study. Carrier state for CD was confirmed in additional two patients who were evaluated for suspected PID because of recurrent otitis media and recurrent skin abscesses. Cumulatively, 5/24 (21%) of CD carriers had characteristic infections by the end of the study.

### Mortality in CD

None of the patients or carriers of CD from the Slovenian PID registry died because of CD; however, three relatives of registered patients died because of clinical manifestations possibly related to CD. A sister of a patient with C2 deficiency died from *H. influenzae* type b meningitis at the age of 2 months; two uncles of patients with HAE died from laryngoedema (one during dental procedure). Since these relatives were not evaluated for PID they were not included in the national PID registry.

### Comparison of Results of Functional Complement Assays Between Subjects With Homozygous or Compound Heterozygous *C2* or *C8* Mutations and Carriers of Heterozygous Mutation Causing C2 or C8 Deficiency

**Figure 2:**
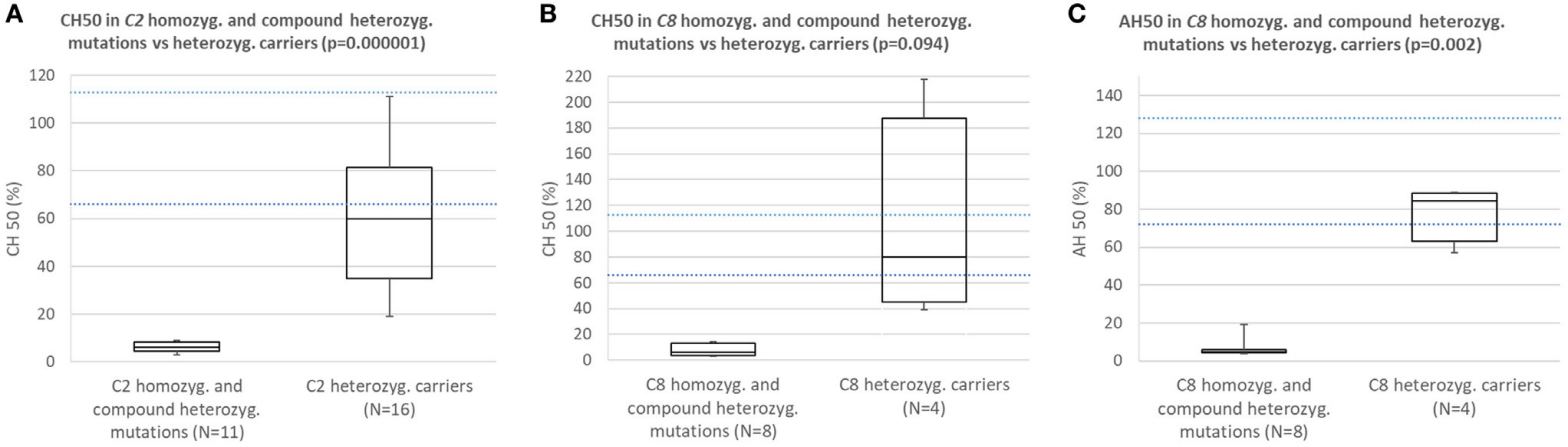
**(A)** Comparison of CH50 between subjects with homozygous or compound heterozygous mutations in *C2* and heterozygous carriers of C2 deficiency. **(B)** Comparison of CH50 between subjects with homozygous mutations in *C8* and carriers of heterozygous mutation causing C8 deficiency. **(C)** Comparison of AH50 between subjects with homozygous mutations in *C8* and carriers of heterozygous mutation causing C8 deficiency.

Subjects with homozygous or compound heterozygous mutations in *C2* consistently had CH50 below the lower normal limit and significantly lower CH50 than carriers of heterozygous mutation causing C2 deficiency (Figure 2A; *p* = 0.000001).

Subjects with homozygous mutations in *C8* consistently had CH50 and AH50 below lower normal limit and significantly lower AH50 than carriers of heterozygous mutation causing C8 deficiency (Figures 2B,C; *p* = 0.094 for CH50; *p* = 0.002 for AH50).

In five subjects with C6, C7 and combined C6/C7 CD average CH50 was 7%, while average AH50 in these and one patient with properdin deficiency was 14%.

### Comparison of Results of Functional Complement Tests and Concentrations of Affected Complement Components Between (a) Patients With Complete CD With Infections; (b) Subjects With Complete CD Without Infections; (c) Patients With Partial CD With Infections; (d) Subjects With Partial CD Without Infections (Patients With HAE Are Excluded)

**Figure 3:**
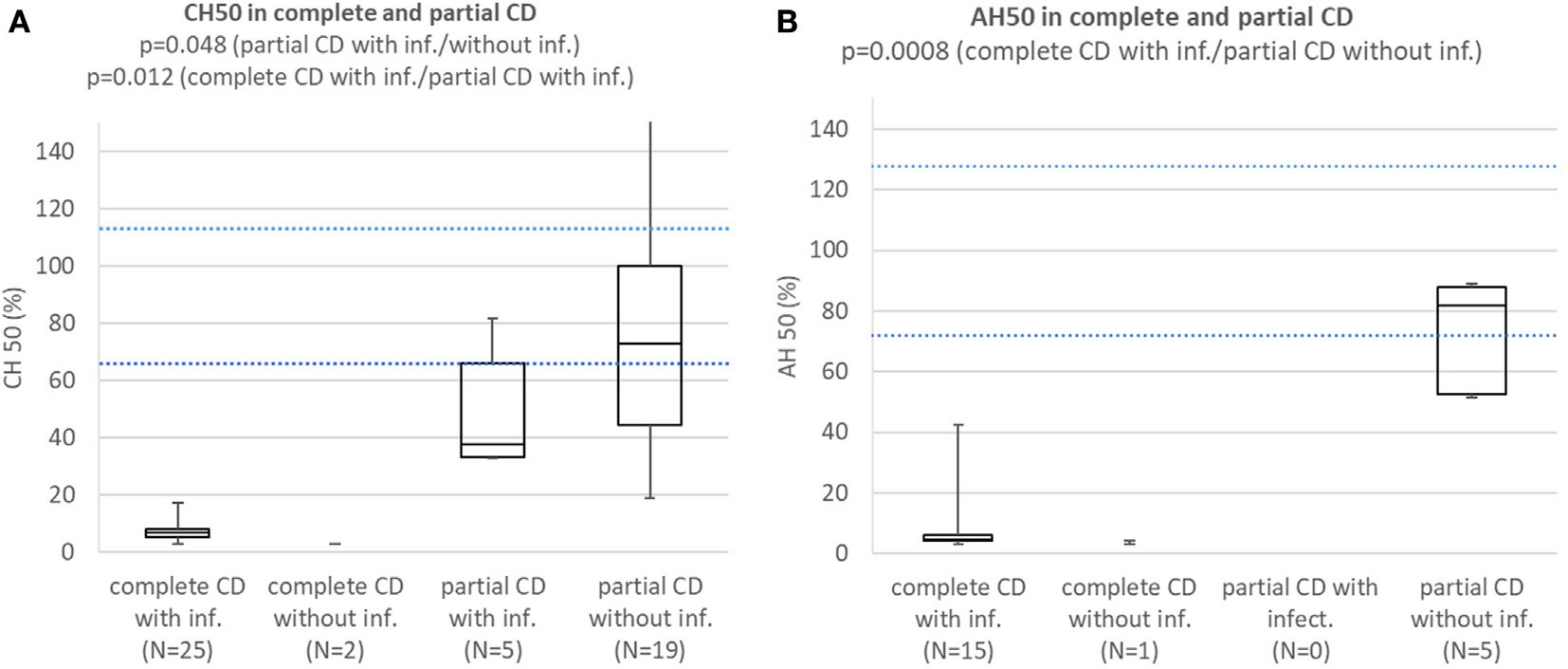
**(A)** Comparison of CH50 between patients with complete CD with infections, complete CD without infections, patients with partial CD with infections, and subjects with partial CD without infections. Patients with hereditary angioedema (HAE) and properdin deficiency are excluded. **(B)** Comparison of AH50 between patients with complete CD with infections, complete CD without infections, patients with partial CD with infections, and subjects with partial CD without infections. Patients with HAE and CD not affecting AH50 are excluded.

Patients with complete CD with infections consistently had CH50 below lower normal limit and significantly lower than subjects with partial CD with (*p* = 0.012) or without infections (*p* = 0.000002). Patients with partial CD with infections had significantly lower CH50 than subjects with partial CD without infections (*p* = 0.048) (Figure 3A). Patients with HAE and properdin deficiency were excluded. Patients with complete CD with infections consistently had AH50 below lower normal limit and significantly lower than subjects with partial CD without infections (*p* = 0.0008) (Figure 3B). Patients with HAE and CD not affecting AH50 were excluded.

**Figure 4:**
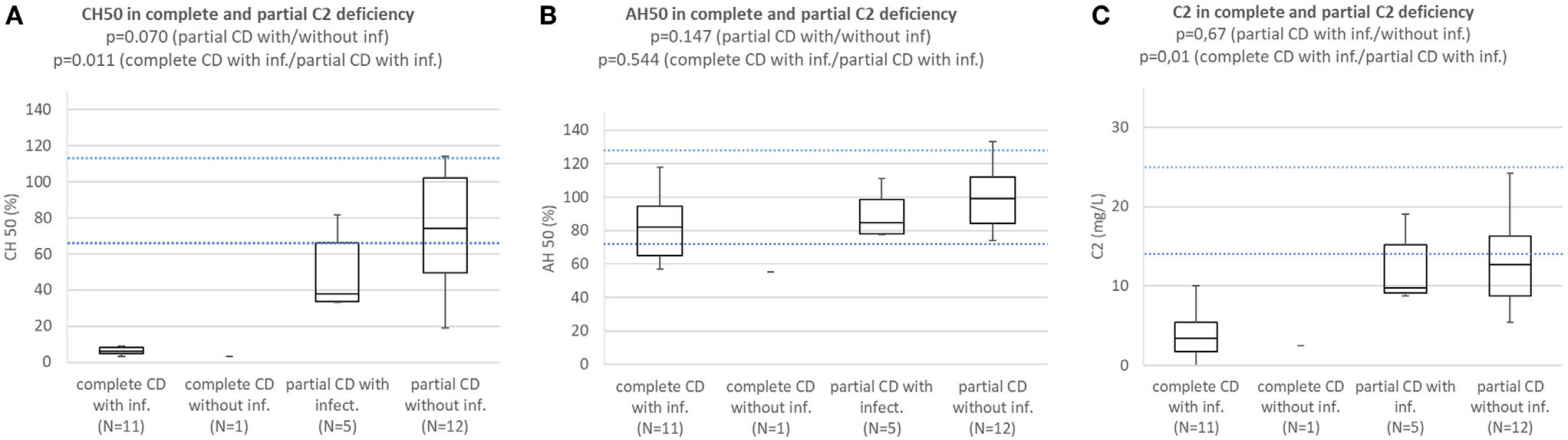
**(A)** Comparison of CH50 between patients with complete C2 deficiency with infections, complete C2 deficiency without infections patients with partial C2 deficiency with infections, and subjects with partial C2 deficiency without infections. **(B)** Comparison of AH50 between patients with complete C2 deficiency with infections, complete C2 deficiency without infections, patients with partial C2 deficiency with infections, and subjects with partial C2 deficiency without infections. **(C)** Comparison of C2 concentration between patients with complete C2 deficiency with infections, complete C2 deficiency without infections, patients with partial C2 deficiency with infections, and subjects with partial C2 deficiency without infections.

Patients with complete C2 deficiency with infections consistently had CH50 below lower normal limit and significantly lower than subjects with partial C2 deficiency with (*p* = 0.011) or without infections (*p* = 0.000001). Difference in CH50 between patients with partial C2 deficiency with infections and subjects with partial C2 deficiency without infections was not significant (*p* = 0.070) (Figure 4A). Patients with complete and partial C2 deficiency had median AH50 within normal limits and differences between subgroups were not significant (Figure 4B). Patients with complete and partial C2 deficiency had median C2 concentration below normal limits regardless of presence of infections. Patients with complete C2 deficiency had significantly lower C2 concentration than both patients with partial C2 deficiency with infections (*p* = 0.01) and subjects with partial C2 deficiency without infections (*p* = 0002); however, the difference in C2 concentration between patients with partial C2 deficiency with infections and subjects with partial C2 deficiency without infections was not significant (*p* = 0.67) (Figure 4C).

**Figure 5:**
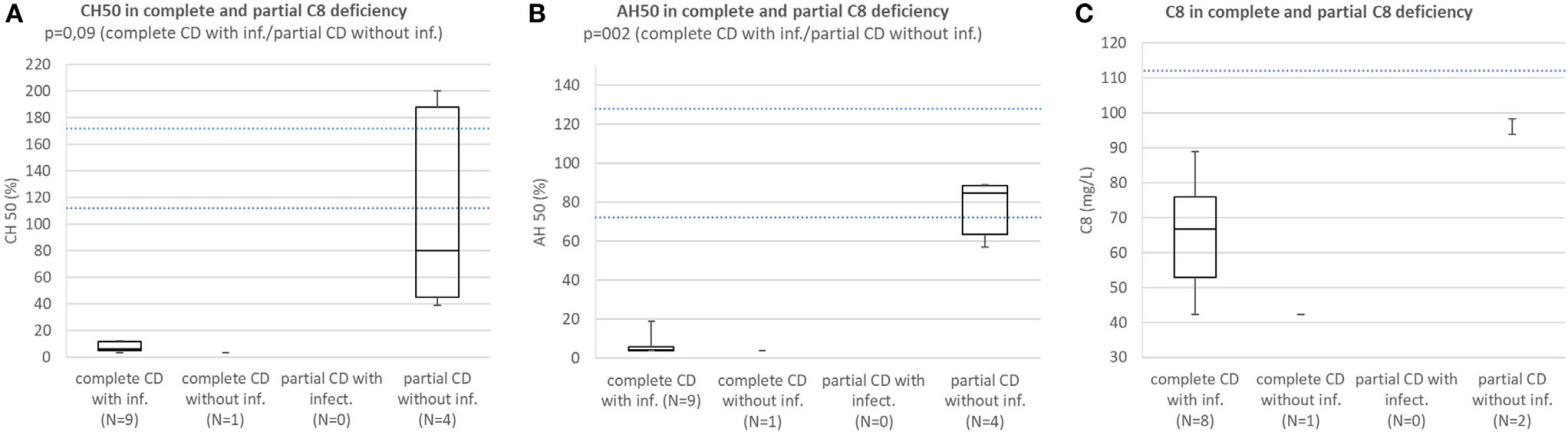
**(A)** Comparison of CH50 between patients with complete C8 deficiency with infections, complete C8 deficiency without infections, patients with partial C8 deficiency with infections, and subjects with partial CS deficiency without infection. **(B)** Comparison of AH50 between patients with complete C8 deficiency with infections, complete C8 deficiency without infections, patients with partial C8 deficiency with infections, and subjects with partial C8 deficiency without infection. **(C)** Comparison of C8 concentration between patients with complete C8 deficiency with infections, complete C8 deficiency without infections, patients with partial C8 deficiency with infections, and subjects with partial CS deficiency without infection.

Patients with complete C8 deficiency with infections consistently had CH50 below lower normal limit. Median CH50 of subjects with partial C8 deficiency without infections was below lower normal limit and the difference in CH50 between patients with complete C8 deficiency with infections and subjects with partial C8 deficiency without infections was not significant because of low sample size (*p* = 0.094) (Figure 5A).

Patients with complete C8 deficiency with infections had median AH50 and C8 concentration below lower normal limit; however, subjects with partial C8 deficiency without infections had AH50 close to lower normal limit and C8 concentration below lower normal limit. However, the difference in AH50 between the groups was significant (*p* = 0.002) (Figures 5B,C).

### Novel Mutations in *C2* Gene

Two novel mutations in *C2* gene were identified in this study. The first was heterozygous substitution NM_000063: c.1754A>C in exon 14 which changes conserved amino acid threonin at position 585 into methionine (NP_000054.2 p.Thr585Met). Mutation pathogenicity was predicted through several *in silico* prediction tools: SIFT (deleterious 0.011), Polyphen2 (probably damaging 0.9), CADD score (33), and Mutation taster (disease causing 0.999). Variant is not present in dbSNP, ExAC, and gnomAD databases. All three subjects with this mutation who were also heterozygous for common 28 bp deletion, had severely reduced CH50 to the range of two homozygous pathogenetic mutations.

The second novel mutation found in exon 16 is a heterozygous missense mutation NM_000063: c.2024G>T which changes conserved amino acid cysteine at position 675 into phenylalanine (NP_000054.2 p.Cys675Phe). Mutation pathogenicity was predicted with several *in silico* programs: SIFT (deleterious 0.016), Polyphen2 (possibly damaging 0.902), CADD score (30), and Mutation taster (disease causing 0.999). Variant is not present in dbSNP, ExAC and gnomAD databases. We have identified only one carrier of this mutation who had reduced both average CH50 (38%) and C2 concentration (10 mg/L) to the range that would be expected for a carrier of a pathogenetic mutation in *C2*.

### Comparison of CH50 Between Specific Mutations in *C2*

**Figure 6:**
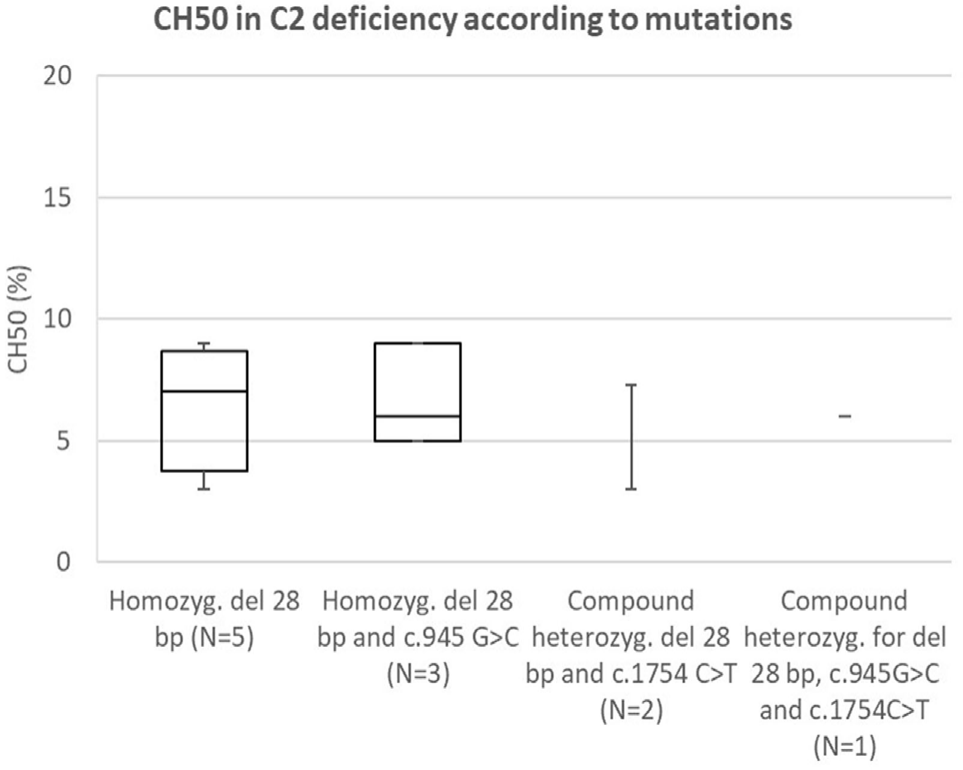
Comparison of CH50 between subjects with C2 deficiency, according to underlying genetic defect.

Analysis of correlation between known pathogenetic mutation (28 bp deletion) and novel mutation (c.1754A>C) in *C2* (presented in Figure 6) showed comparable reduction in CH50 in both mutations. Another novel mutation in C2 (c.2024G>T) was not present in homozygous form; however, average CH50 in a carrier of this mutation was 38%, a reduction consistent with pathogenetic heterozygous mutation (see Figure 2A).

### Combined C6/C7 Deficiency

At the time of the study, there was only one patient with combined C6 and C7 deficiency included in the Slovenian PID registry. Subtotal C6 deficiency was previously reported, in some cases combined with subtotal C7 deficiency (13).

### C1 Inhibitor Function and Concentration in Patients With HAE

The median C1 inhibitor concentration and function were 0.188 mg/L (range 0.048–0.66) and 16% (range 1–64%), respectively. Concentration of C4 was 0.089 mg/L (range 0.048–0.154).

## Discussion

In this study, we conducted analysis of prevalence of CD and demographic characteristics of patients with CD in Slovenia, with special focus on correlation of clinical manifestations with complement laboratory analysis at functional, protein, and genetic levels. Despite high prevalence of mutations in complement genes [the most common are mutations in C2 with a prevalence about 1:20,000 individuals (14)], CD represent only a minority of all PID in most national PID registries. Only 16 out of 3,083 (0.5%; year 2009), 13 out of 1,368 (0.9%; year 2012), 4 out of 147 (2.7%; year 2010), and 16 out of 348 (4.6%; year 2014) patients with CD were registered in the French (15), German (16), Greek (17), and Swiss (18) national PID registries, respectively. Low prevalence of CD was observed in the ESID registry as well. The percentage of CD increased from 0.5% of 1,173 patients in 2005 to 4.9% of 19,091 patients in 2014 (https://esid.org/Working-Parties/Registry/ESID-Database-Statistics). Prevalence of HAE in Slovenia (1:80,000) is similar to other reports (1:50,000) (8, 9). In contrast to low prevalence of CD in other reports, the frequency of CD is significantly higher in the Slovenian PID registry, where 26% (68 of 257) of all patients with PID represent patients with CD. Functional complement tests can be temporary reduced in subjects without CD because of complement consumption or improper transport; therefore, only patients with persistently reduced functional complement tests were included in the Slovenian PID registry, which increases reliability of epidemiologic PID data. Diagnosis of CD was genetically confirmed in all patients with CD in the registry for whom genetic testing was possible (45% of all registered patients with complement component deficiency) and in 96% of patients with HAE. True prevalence of CD, as calculated from genetic studies in unselected populations, is at least 10 times higher than reported (14), meaning that there could still be unrecognized patients with CD. Low number of recognized patients with complement component deficiency could be a consequence of under-recognition because patients with bacterial infections of the respiratory system and autoimmunity are not evaluated for CD. High percentage of neisserial (47% had *N. meningitidis* infection before CD diagnosis) in the European CD cohort probably reflects that complement analysis is regularly performed only after neisserial infections (6). Unavailability of laboratory facilities to determine classical and alternative pathways and problems related to *in vitro* complement activation due to inappropriate handling of samples during transport limit complement evaluation as well (19). Another reason for under-recognition could be absence of infections until adulthood in some patients with CD (20). In addition, patients with CD vaccinated against encapsulated bacteria (*S. pneumoniae, H. influenzae* type b, *N. meningitidis*) might not be diagnosed with CD, because these characteristic infections would be prevented (21). However, delay in diagnosis of CD is associated with increased morbidity and an older age at diagnosis is significantly associated with mortality (20). Therefore, awareness campaigns about CD in PID community should be promoted to improve timely recognition of CD worldwide. The number of laboratories performing complement function tests should increase to improve the availability for screening more patients with infections with encapsulated bacteria and autoimmune disease. Health-care providers should be aware that handling of blood samples, including collection and storage are critically important for some of the complement assays (19).

Correct identification of subjects with CD with increased risk for clinical manifestations is crucial as it is possible to significantly lower the risk of severe respiratory infections, sepsis and meningitis with vaccination against *S. pneumoniae, H. influenzae* type b, and *N. meningitidis* and shorten delay of diagnosing autoimmune disease. In contrast to the European cohorts of CD, where CD was usually diagnosed after bacterial meningitis (6, 22), complement component deficiency (especially C2 deficiency) was diagnosed in many Slovenian patients after recurrent bacterial infections of respiratory system (Table 1). The presenting infectious features of 23 Slovenian patients with C2, C6, C6/7, C8, and properdin deficiencies were pneumonia (10 pt) sepsis (9 pt), purulent meningitis (7 pt), otitis (4 pt), cellulitis (2pt) and impetigo, sinusitis, and herpes zoster each in 1 patient (Table 1). These results implicate that limiting complement evaluation to patients with meningitis could account for under-recognition of CD. It should be stressed that many infections in our cohort were recurrent (pneumonia 6 pt, otitis 4 pt, sepsis 2 pt, purulent meningitis 2 pt, herpes zoster 1 pt).

Not only patients with infections but also patients with autoimmunity should be screened for CD as C2 deficiency is detected in about 1% of Caucasian patients with SLE (23). However, autoimmunity was observed only in 1 out of 17 patients with genetically confirmed C2 deficiency in Slovenian PID registry. CD was diagnosed when the patient was evaluated for glomerulonephritis presenting in infancy, 2 weeks after vaccination against diphtheria, tetanus, pertussis, polio, and *H. influenzae* type b.

Laryngeal edema is an important cause of mortality in patients with HAE; therefore, relatives of patients with HAE should be evaluated for HAE before life-threatening complications occur.

### The Power of Functional Complement Tests in Differentiating Complete and Partial CD

In the past decades, identification of CD was only possible with functional complement tests, but nowadays more subjects with CD are expected to be diagnosed through next generation sequencing. Therefore, it is important to know the correlation between mutations in complement system, complement functional studies and risk for infections in subject with CD. Our study confirmed previous reports that results of complement functional tests differentiate subjects with homozygous mutations in complement genes from heterozygous carriers (19).

Additional strength of the present study is the finding that functional tests can predict the risk for characteristic infections even in subgroups of subjects with partial reduction of complement (carriers of heterozygous mutation causing mutation in CD genes). Patients with partial CD with characteristic infection had significantly lower CH50 than subjects with partial CD without infections. This was not observed in previous studies, where heterozygous carriers of CD in general did not have significantly increased risk of infections (5, 20). In our patients, complement functional studies better correlated with characteristic infections than concentrations of affected complement component; therefore, we suggest performing CH50 and AH50 tests in all subjects with CD and protection with vaccines to all subjects with more than borderline reduction of complement functional studies.

### Novel Mutations

Patients with novel c.1754C>T mutation had profoundly reduced CH50 that was not different to CH50 in patients with known deleterious homozygous deletion of 28bp. The carrier of another novel mutation c.2024G>T had reduced both average CH50 and C2 concentration to the range that would be expected for a carrier of a pathogenetic mutation in *C2*.

However, normal C2 concentration was observed in a family member with compound heterozygous mutations c.945G>C and c.1754C>T, which is consistent with previous observation that c.945G>C mutation is a non-deleterious polymorphism (4, 24).

## Conclusion

Complement deficiency may be widely under-recognized and should be suspected in any patient with recurrent or severe infections with encapsulated bacteria and in patients with autoimmunity, especially SLE and glomerulonephritis. This study showed that complete CD predicts homozygous or compound heterozygous deleterious mutations in complement genes, while partial CD predicts heterozygous carrier state. Furthermore, risk for characteristic infections can be predicted according to the results of complement functional tests even in carriers of heterozygous mutation causing CD. Observed prevalence of characteristic infections in heterozygous CD carriers with reduced complement activity supports active protection through vaccination against encapsulated bacteria not only in patients with CD but also in subjects with partial CD. Screening of family members of subjects with CD is recommended not only for patients with deficiency of complement components, but also in patients with HAE, because morbidity and mortality in patients with HAE can be reduced with available preventive measures, especially before surgical procedures close to airway.

## Ethics Statement

This study was carried out in accordance with the recommendations of National Medical Ethics Committee with written informed consent from all subjects. All subjects gave written informed consent in accordance with the Declaration of Helsinki.

## Author Contributions

ŠB contributed conception and design of the study and performed statistical analysis; ŠB and MK organized the database; ŠB, MK, MD, and SSimčič wrote the first draft of the manuscript; ŠB, MK, GM, PK, BZ, MP, and TA performed clinical evaluation of patients; SSimčič and SStopinšek performed functional analysis of complement; MD performed genetic analysis in complement genes. All authors contributed to manuscript revision, read and approved submitted version.

## Conflict of Interest Statement

The authors of this manuscript declare that work was carried out in the absence of any personal, professional, or financial relationships that could potentially be construed as a conflict of interest.
