# Supplementary material for: Functional Complement Analysis Can Predict Genetic Testing Results and Long-Term Outcome in Patients With Complement Deficiencies
Source: Front Immunol. 2018 Mar 21;9:500. doi: 10.3389/fimmu.2018.00500 (PMC5871747; doi:10.3389/fimmu.2018.00500)
Supplement: Supplementary file 1 [file data_sheet_1.docx]

**Supplementary Methods**

**Functional complement assays and concentrations of complement components**

The activity of the classical complement pathway (CH50; normal range: 63-137%) and the alternative pathway (AH50; normal range: 77-123 U) was determined by hemolytic titration assays, where dilutions of patient’s serum are incubated with antibody-sensitized sheep erythrocytes or rabbit erythrocytes and results are expressed as percentage of a pooled normal human serum. Serum concentrations of complement components C2 (normal range: 14-25 mg/l), C6 (normal range: 45-96 mg/l), C7 (normal range: 55-85 mg/l) and C8 (normal range: 112-172 mg/l) were measured by radial immunodiffusion (The Binding Site, UK). Only measurements of complement components and functional complement assays in patients after the first year of age were taken into account, as they are sometimes severely, but transiently reduced in healthy infants.(1,2) Serum protein concentration of C1 inhibitor (normal range: 0.20–0.35 g/l), C4 (normal range: 0.16–0.31 g/l) (Siemens, Marburg, Germany) was quantified by means of radial immunodiffusion and C1 inhibitor activity (functional levels ≤40% of normal are considered decreased) was measured using enzyme immunoassay (Quidel Corporation, CA, USA) in concordance to the manufacturer's instructions.

**Genotyping in C1-C9 deficiencies**

**DNA isolation and primer selection**

Peripheral blood (5 mL) for DNA isolation was taken together with blood for routine laboratory work. DNA isolation was performed using the FlexiGene isolation kit (Qiagen, Germany), according to the recommended protocol. DNA was stored at the 4 °C prior to further molecular analyses. PCR primers (Eurofins MWG Operon, Germany) were designed according to the established laboratory protocol (sequences are available upon request), covering whole coding region and intron/exon boundaries of whole *C2* gene (18 exons) and exon 9 of *C8B* gene.

**Polymerase chain reaction**

The reaction with GoTaq G2 Green Master Mix (Promega, USA) was set up using 100 ng of genomic DNA and 2X GoTaq G2 Green Master Mix in a final reaction volume of 20 μL. The thermocycling procedure consisted of initial denaturing step at 95°C for 2 min followed by 35 cycles of 94°C for 30 s, annealing step at 58°C for 30s, extension step at 72°C for 40 s and final extension at 72°C for 7 min. The PCR products were subjected to electrophoresis in 1.5% agarose gel stained with SyberGreen and photographed under ultraviolet light using the UV G:BOX (Syngene, USA).

**Sanger sequencing**

PCR products (2,5 μL) were purified using 1 μL ExoSAP-IT enzyme mix (Affymetrix, USA), (incubation at 37°C for 15 min, enzyme deactivation at 80°C for 15 min) and subjected to direct nucleotide sequencing using the Big Dye Terminator cycle sequencing kit (Applied Biosystems, USA) following the manufacturer’s instructions. The sequencing was performed on Applied Biosystems 3500 Genetic Analyzer (Applied Biosystems, USA). Sequences were analyzed with the Sequencing Analysis Software v5.2.0 (Applied Biosystems, USA) and alignment was performed with the Nucleotide BLAST program (<https://blast.ncbi.nlm.nih.gov/Blast>). All identified mutations/variants were validated in additional independent round of PCR and once again sequenced.

Data from the Sequence Analysis Software were aligned with the native *C2* sequence (NM_000063) and *C8B* sequence (NM_000066), (<http://www3.ncbi.nlm.nih.gov>). Nomenclature is cited according to the HGVS guidelines ([www.hgvs.org/mutnomen](http://www.hgvs.org/mutnomen)): ‘c.’ indicates a coding DNA sequence and ‘p.’ indicates a protein sequence. Sequence variants were checked using the Mutalyzer program (http://www.LOVD.nl/mutalyzer). Family segregation analysis was performed in several family members. All genetic analyses for *C2* and *C8B* deficiency were performed in the genetic laboratory of the University Children’s Hospital Ljubljana, Slovenia.

**Genotyping in HAE**

Genomic DNA was extracted from EDTA-containing whole blood samples using a QIAamp DNA Blood Mini Kit (Qiagen, Hilden, Germany) per the manufacturer's instructions. The detection of SERPING1 mutations, in promoter, noncoding exon 1 and in the 7 coding exons and exon-intron boundaries were performed as described previously(3,4). To identify mutations, all sequences were compared with the SERPING1 reference sequence in the GenBank (GenBank accession number # X54486.1). Alignment was performed with the Nucleotide BLAST program (<https://blast.ncbi.nlm.nih.gov/Blast>).

References

1. Johnson U, Truedsson L, Gustavii B. Complement components in 100 newborns and their mothers determined by electroimmunoassay. *Acta Pathol Microbiol Immunol Scand C* (1983) **91**:147–50.

2. Sonntag J, Brandenburg U, Polzehl D, Strauss E, Vogel M, Dudenhausen JW, Obladen M. Complement system in healthy term newborns: reference values in umbilical cord blood. *Pediatr Dev Pathol* **1**:131–5.

3. Bowen B, Hawk JJ, Sibunka S, Hovick S, Weiler JM. A review of the reported defects in the human C1 esterase inhibitor gene producing hereditary angioedema including four new mutations. *Clin Immunol* (2001) **98**:157–63. doi:10.1006/clim.2000.4947

4. Speletas M, Boukas K, Papadopoulou-Alataki E, Tsitsami E, Germenis AE. Hereditary angioedema in Greek families caused by novel and recurrent mutations. *Hum Immunol* (2009) **70**:925–9. doi:10.1016/j.humimm.2009.08.010
